# Effect of* Rheum Ribes *Hydro-Alcoholic Extract on Memory Impairments in Rat Model of Alzheimer's Disease

**Published:** 2015

**Authors:** Maryam Zahedi, Mohammad Reza Hojjati, Hossein Fathpour, Zahra Rabiei, Zahra Alibabaei, Arezoo Basim

**Affiliations:** a *Department of Biology, Shahrekord Branch, Islamic Azad University, Shahrekord, Iran.*; b *Physiology Department, Medical Facutly, Shahrekord University Of Medical Sciences, Shahrekord, Iran. *; c *Medical Plants Research Center, Shahrekord University of Medical Sciences, Iran.*

**Keywords:** Rat model of Alzheimer's disease, *Rheum ribes*, Shuttle box, Spatial learning, Memory

## Abstract

Some animal models have been used to study Alzheimer's disease (AD). AD is an irreversible progressive neurodegenerative disease and the most common cause of dementia. Animal studies have shown that there is a relation between decrease in cholinergic functions in the nucleus basalis of Meynert (NBM) and loss of learning capability and memory. The aim of this study was to investigate the effect of *Rheum ribes* extract (RR) on memory deficit in one of the rat models of AD. Plant (1500gr) was collected from Saman (kahkesh) region of Chaharmahal Va Bakhtiari province in Iran. RR hydro-alcoholic extracts were prepared using maceration method. Rat model of Alzheimer was induced by Nucleus Basalis of Meynert lesions (NBML). Animals (n = 32) received extracts for 20 days and then passive avoidance and Morris water maze tasks were performed for memory evaluation. FRAP and HPLC methods were used for measurement of the antioxidant and Malondialdehyde (MDA) levels in blood. In water maze experiment, probe trial results showed that NBML group spent significantly less time in target quadrant, in which the platform was located on the preceding day. In addition, the time spent in target quadrant was significantly increased in NBML + RR groups (250 and 500 mg/kg) compared to the NBML group. In passive avoidance task, mean initial latency time and step-though latency were significantly decreased in NBML group. RR extracts significantly prolonged step-through latency in NBML + RR groups. Results of this study suggest that *Rheum ribes *extracts can improve memory deficits induced by bilateral NBM lesions in rats.

## Introduction

Alzheimer's disease (AD) is the most common cause of dementia. This irreversible progressive neurodegenerative disease occurs gradually and results in memory loss, abnormal behavior, personality changes, and a decline in thinking abilities (1). It is estimated that the annual incidence of Alzheimer disease increases dramatically with age, from approximately 53 new cases per 1000 people age 65 to 75, to 170 new cases per 1000 people age 75 to 84, to 231 new cases per 1000 people age 85 and older (2). Pathophysiology of AD includes development of plaques around the neurons and hyper-phosphorylated tau protein inside the neurons which are the reasons for cognitive ability loss in the severe and end stage of AD (3). Neuropathology of AD consists of loss of basal forebrain cholinergic neurons which leads to decreased cholinergic transmission. This can be corrected with acetylcolinesterase enzyme (4). Plaques are detected in extensive areas of the brain, including cortex, hippocampus, basal ganglia, thalamus and even cerebellum (5).

The fact that the basal forebrain, particularly the NBM, degenerates in AD has excited investigators to try to model this property in experimental animals by a variety of lesioning methods (6). There is a high percent reduction in cholinergic neurons in the NBM while the disease is in progress. Animal studies show that there is a relation between decrease in cholinergic functions in this areas and loss of learning and memory capabilities (7). It has been shown that anticholinergic medications and NBM lesions lead to loss of learning and memory capabilities using water maze and passive avoidance learning tasks (8). Attempts have been made to lessen the decrease of acetylcholine level in the brain of patients with learning and memory impairments using medications, such as precursors of acetylcholine, muscarinic and nicotinic antagonists and acetylcholinesterase inhibitors. Meanwhile, the use of acetylcholinesterase inhibitors is considered to be the most important strategy of treatment. Based on these finding, consequently medications, such as tacrine, donepezil, galantamine and rivastigmine have been introduced to the market (7). However; because of the side effects of these medications, such as weakness, anorexia, nausea, muscle cramps, cardiovascular and respiratory disorders, researchers are recently looking for herbal medications with fewer side effects (9). Oxidative damage is a major player in neural degeneration and several studies have demonstrated that in early occurring condition of AD oxidative stress has major role. Antioxidants act by removing and scavenging reactive oxygen species (ROS), and its precursors, and binding to metal ions necessary for the catalysis of ROS generation (10).

*Rheum ribes* (RR, Rhubarb) is one of the medicinal herbs which belongs to the family of Polygonacea (11). This plant is native to some parts of Asia, including Iran, Pakistan, India, China and Turkey (11). In some parts of Iran *Rheum ribes* is used as a kind of food, making jam, laxative and hair dyeing (12). In alternative medicine, boiled stems and dried roots of this plant are used for the treatment of anemia, anorexia, weakness, mental fatigue, diabetes, infectious abscess, gangrene, hypertension, obesity and wound (13). Sayyah and colleagues have also mentioned that hydro-alcoholic extract of RR is effective in the treatment of depression (14) and obsessive compulsive disorders (15). In Iranian traditional medicine, this plant is used as sedative and mood enhancer which suggests its effect on central nervous system (15). “Rhubarb was first recorded and rated as one of the inferior remedies in the oldest Chinese herbal books” (16). Traditional Chinese medicine suggests Rheum ribes enhances the memory in old patients (17). Extract of root and stem of RR has high antioxidant activity (18). Epidemiological and laboratory findings reveal that foods containing antioxidants delay the progress of Alzheimer's disease probably due to prevention or neutralization of detrimental effects of free radicals (19). This herb contains potassium, iron, zinc, selenium, flavonoid, phenol, quercetin and remarkable amount of vitamins A, C and E (20, 21). It also contains Chrysophanol, Rhein and some glucosides (15). Acetylcholinesterase inhibitory property of this plant is also important. Acetylcholinesterase inhibitory properties of this plant ranked third among 100 species which are native to Iran (9). Therefore; it seems that Rheum ribes extract can have positive effect on memory impairments in rat model of Alzheimer. In our previous study, we evaluated the effect of other traditional herb (*Cyperus rotundus*) on learning and memory deficits using same method of lesions in NBM nuclus (8). In this study, we investigated the effect of RR hydro-alcoholic extract on spatial memory and passive avoidance learning in rat model of Alzheimer's disease in which their NBM nucleus is bilaterally damaged.

## Experimental

*Preparation of the extract*

*Rheum ribes *(1500 gr) was collected from Saman (kahkesh) region of Chaharmahal Va Bakhtiari province in Iran (Latitude: 32º27'36.54" N, Longitude: 50º55'6.32" E, Height: 1921.15 m). The sample was identified by experts at the Medical plant center, Medical Faculty, Shahrekord University of Medical Sciences. A voucher specimen (SKUMS No. 318) has been deposited in the herbarium of Medical Faculty, Shahrekord University of Medical Sciences. Roots and rhizomes were separated, washed and ground. To prepare hydro-alcoholic extract, 250 g of the ground herb was macerated in two liters of methanol and water (1 liter of each) for four days. Then it was filtered and concentrated under reduced pressure using a rotary apparatus. Remaining contents were transferred into Petri dishes and were put into an oven with 37ºc temperature to be dried up. The powdered extract was dissolved in water and used when it was needed (21).

*Determination of radical scavenging activity of Rheum ribes extract*

Determination of Radical scavenging activity of RR extract was determined according to Moon and Terao (22) with a slight modification based on ability to scavenge 2, 2-diphenyl-1-picrylhydrazyl (DPPH) stable radicals. Briefly, various concentrations of the extract (5-60 µg/ml) were mixed with DPPH solution in ethanol (2 ml). The reaction mixture was shaken at room temperature in a dark room. After 35 min at room temperature, the absorbance was recorded at 517 nm using a UV-Vis spectrophotometer (Biochrom Ltd, England) (22). Butylated hydroxytoluene (BHT) was used as a positive control.

IC _50_ indicates the concentration of the extract which neutralizes 50% of free radicals of DPPH and is stated as the inhibitory percentage of extract. Inhibition of free radical by DPPH (%) was calculated as follows:

I (%) = 100 × (A _control _- A _sample_)/ A _control_

*Determination of total phenolic content of Rheum ribes extract*

Total phenolic content was determined as described by Singlton (23) with minor modification. 500 µl extract solution (1 mg extract in 10 ml ethanol) was added to 5 ml Folin-Ciocalteu reactive (10%) and after 3 min. shaking, 3 ml of Na_2_CO_3_ (2%) was added and was shaken again. After 1 h at room temperature, absorbance was measured at 760 nm. Gallic acid was used as the standard for the calibration curve. Total phenolic content was calculated as µg Gallic acid equivalent (GAEq) by using the following linear equation according the calibration curve. The result is average of triplicate analyses.

Absorbance = 0.0039 (GAEq) + 0.0035

*Animals*

Male Wistar rats, (age: 8 weeks, weigh: 250-300 g), were purchased from Pasteur institution (Tehran, Iran). Rats were housed in groups of four at 25ºC with controlled 12/12-h light/dark cycle. Food and water were freely available. All experiments were executed in Spring season (3 Months) and were conducted in accordance with the Guide for the Care and Use at Laboratory Animals and were approved by Research and Ethics Committee at Shahrekord University of Medical Sciences. Animals were assigned randomly into six different groups with eight rats in each group:

Group 1: Control group received distilled water (i.p.) without surgery.

Group 2: NBM lesioned rats (NBML) in which both Nucleus Basalis of Meynert were bilaterally destroyed and received distilled water (i.p.) after recovery from the surgery.

Group 3 and 4: NBML + RR (250, 500) in which NBM nuclei were bilaterally destroyed and then received the *Rheum ribes *extracts (250 and 500 mg/kg, i.p., respectively) for 20 days after recovery from the surgery.

Groups 5: and 6: Intact + RR (250, 500), normal animals which received *Rheum ribes *extracts (250 or 500 mg/kg, i.p., respectively) for 20 days without any surgery. Doses were chosen based on previous studies (24).

*Stereotaxic Surgery*

Animals were anesthetized using ketamine hydrocholoride (110 mg/kg, i.p.) and xylazine (4 mg/kg, i.p.) injections. Rats were implanted with a twisted bipolar stainless-steel electrode (Plastic Products MS 301/1, 0.25 mm in diameter; Bilaney, Düsseldorf, Germany) in one hemisphere under conventional stereotaxic procedures. The electrode was conducted into the NBM with the incisor bar set at -2.7 mm below the interaural line and according to the following coordinates from the stereotaxic atlas of AP: –1.30 mm from bregma, L: ± 2.8 mm from midline, and DV: -8.00 mm from cranium surface (25). The NBM lesions were made by electrolysis using a current intensity of 2 mA for 15 s. The electrode was withdrawn after induction of lesion at each side. The incision was cleaned and sutured and then the rats were returned to their home cages and allowed 10 days for recovery before behavioral evaluations (6).

*Passive Avoidance Test*

A shuttle box apparatus was used for passive avoidance test. The apparatus consisted one lighted chamber and one dark chamber with grid door. Electrical shocks are transferred by a separated stimulator to grid floor of the shuttle box. This test was performed for each rat for four consequently days. In the first and second days, each rat was put and released in the device to habituate for 60 s. In the third day, an acquisition trial was performed in which animals were initially placed in the lighted compartment and the door between the two compartments was opened 20 seconds later. The initial latency (t1) for a rat to enter the dark compartment was measured. When rat enters the dark compartment, the door was closed and an electric foot-shock (1 mA for 1 s) was delivered through the stainless steel rods with a constant current shock generator. All animals examined, entered the dark compartment within 60 s as cut-off latency in the training session, and received a foot-shock. Step-through latency (t2) for animals was recorded on fourth day using same paradigm, but without foot-shock (7).

*Water Maze Test*

Morris water maze was used for spatial learning and memory assessment. Water maze was located in a room with extra cues (such as clock, poster, table, etc.) around it. Test was performed in a circular tank (diameter, 139 cm; height, 60 cm) which was filled with opaque water (22 ± 1°C) up to 25 cm. A metal round platform with diameter of 10 cm was submerged in the center of one of the quadrants (target quadrant). The place of the platform was in the same position on all trials. A video camera was fixed 1.4 m above the center of the tank and all trials were recorded for later data analysis. For testing learning abilities, animals were trained to perform 2 trials per day (with an inter trial interval of 10 min.) for five consecutive day. In each trial, rats were randomly placed into the water from one of the quadrants and the time from start point to escape onto the platform was measured. Animals had to find the hidden platform within 60 s. If rats could not find the platform, they were led toward the platform to find it. As soon as the rats found the platform, they were let to stay on it for 30s to explore the place of hidden platform using extra cues (26). On the sixth day, rats were individually subjected to a probe trial session for testing the memory by removing the platform and allowing animals to swim for 60 s to search for platform (27).

*Ferric Reducing/Antioxidant Power (FRAP) Assay*

Approximately 5 cc Blood samples were collected from heart of intact rats which had received RR extract and the antioxidant power assay was performed by measuring its ability to reduce Fe ^3+^ to Fe ^2+^ with FRAP (ferric reducing antioxidant power) test according to the procedure described by Benzie and Strain (28). FeSO4 (100 – 1000 µM concentration range) was used as a standard in FRAP assay. The results are average of triplicate analyses and are expressed in µg/ml.

*Measurement of Malondialdehyde (MDA)*

Malondialdehyde has been the most important biomarker in determining lipid peroxidation in the last 30 years (29). The plasma level of MDA was determined as described by Karatas et al. using high performance liquid chromatography instrument (HPLC). Chromatographic determinations were performed on a high-performance liquid chromatography equipped with an 1100 series pump and a UV absorbance detector. An HP 3395 integrator was employed to record retention times, chromatograms, and evaluate peak heights. A technopak 10u C18 reversed-phase column (emission 553 and excitation 515) was used. MDA standards were prepared from 1, 1, 3, 3-tetraethoxypropane.

The optimized assay was carried out as follows: 50 µl plasma or the standard was treated with 50 µl (0.05%) BHT (in absolute ethanol), followed by the addition of 400µl H_3_PO_4_ (0.44 M) and 100 µl TBA (Butylated hydroxytoluene) (42 mM), vortexed and then incubated for 60 min at 100˚C the reaction was stopped by cooling at 4˚C, then 250 μl of n-butanol was added for extraction of MDA-TBA complex. The solution was vortexed and then centrifuged for 5 min at 14 000 rpm to separate two phases. The supernatant (20 µl) was injected into the HPLC system (30). All data are average of triplicate analyses.

*Statistical Analysis*

All the results were expressed as mean ± SE and data was analyzed in SPSS software (ver. 19) using one-way analysis of variance test (ANOVA) followed by post-hoc LSD test. T-test was used for comparing the amount of MDA between control and Alzheimer group. P < 0.05 was considered statistically significant.

## Results and Discussion

*Radical Scavenging Activity of Rheum ribes*

DPP radical scavenging inhibition values for different concentrations of RR extract are shown in Table 1. As seen in the table 1, IC_50_ value was found between concentrations of 20 and 40 µg/ml.

**Table 1:** DPPH radical scavenging activity for various concentrations of *Rheum ribes* hydro-alcoholic extract and BHT as positive control

**Sample**	**Concentration** **(µg/ml)**	**Absorbance after 30min**	**DPP radical scavenging inhibition (I %)** **IC**_50_ ** (µg/ml)**
*Rheum ribes* extract BHT	5 10 20 **40** 60 7.8 15.6 31.2 62.5 125	0.373 0.317 0.248 0.100 0.051 0.634 0.627 0.515 0.471 0.285	10 24 40 **76 (IC**_50_**)** 87 2.5 3.5 20.8 27.5 55.8

IC_50_ value is highlighted in bold.

*Total phenolic content of Rheum ribes extract*

Total Phenolic content of RR hydro-alcoholic extract was found 114.84 ± 4.6 µg/ml.

*Effect of Rheum ribes Extract on Passive Avoiding Learning*

**Figure 1:**
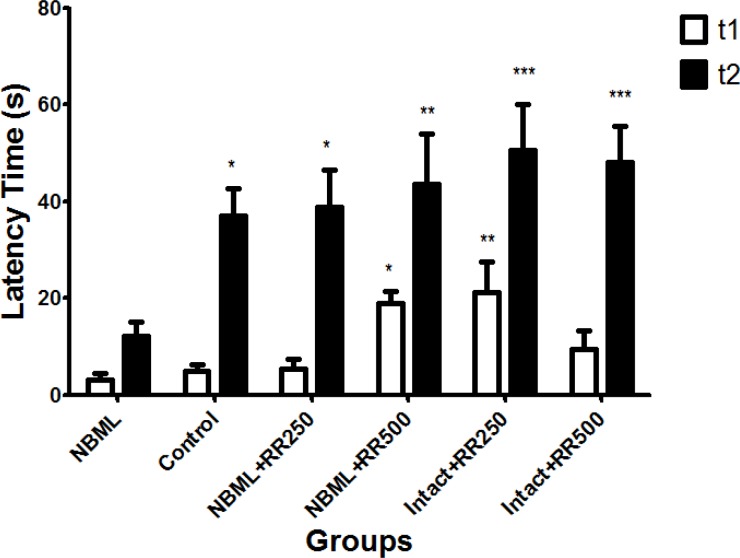
The initial latency (t1) and step-through (t2) latency time in the passive avoidance response. The data are expressed as Mean ± SE; (n = 7 in each group).

As shown in figure 1, mean initial latency (t1) decreased statistically in NBM-lesion group (NBML) when compared with NBML + RR500 and Intact + RR250 groups (p < 0.05 and p < 0.01 respectively). However, no significant difference was seen between NBML, control, NBML + RR250 and Intact + RR500 groups. Step- through latency (t2) reduced markedly in NBML group compared with control group (p < 0.05). T2 latency increased significantly in NBML + RR250 and NBML + RR500 when compared with NBML group (p < 0.05 and p < 0.01, respectively). Both groups of intact animals receiving RR extracts (250 and 500 mg/kg) showed significantly higher step-through latency compared to the NBML group (P < 0.001). In addition, results did not show significant difference for step-through latency time between control group and NBML groups receiving extracts. Moreover, RR extracts (250 and 500 mg/kg) had no remarkable effect in the test session on intact rats when compared with control group (p > 0.05).

* p < 0.05; ** p < 0.01; *** p < 0.001 significant from NBML.

*The Effect of Rheum ribes Extract on Spatial Learning in Water Maze Test*

**Figure 2:**
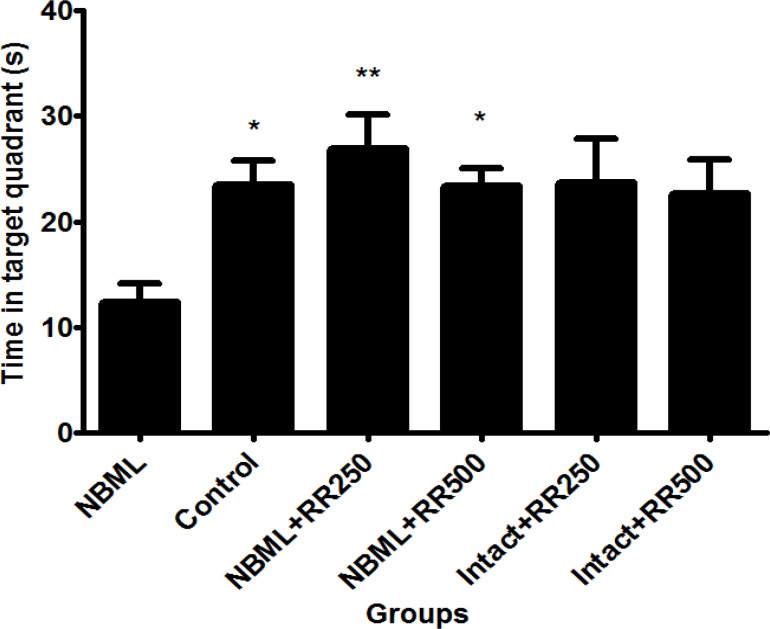
Time spent in the target quadrate in probe trial test. The data are expressed as Mean ± SE; (n = 7 in each group).

Effect of RR extracts on short-term memory was evaluated by performing probe trial experiment using Morris water maze task. Data collected from probe trial experiment revealed that NBM-lesioned rats (NBML) spent significantly less time in target quadrant in which the platform was located in the preceding day compared with control group (P < 0.05, Figure 2). Interestingly, time spent in target quadrant was significantly increased in both NBML groups which received RR extracts (NBML + RR250 and NBML + RR500) when it was compared with the NBML group (p < 0.05 and p < 0.01, respectively). No significant differences were observed between intact animals receiving RR extracts (Intact + RR250 and Intact + RR500) and the control group (p > 0.05).

* p < 0.05; ** p < 0.01 significant from NBML.

*The Effect of Rheum ribes Extract on serum* *Antioxidant level*

**Figure 3:**
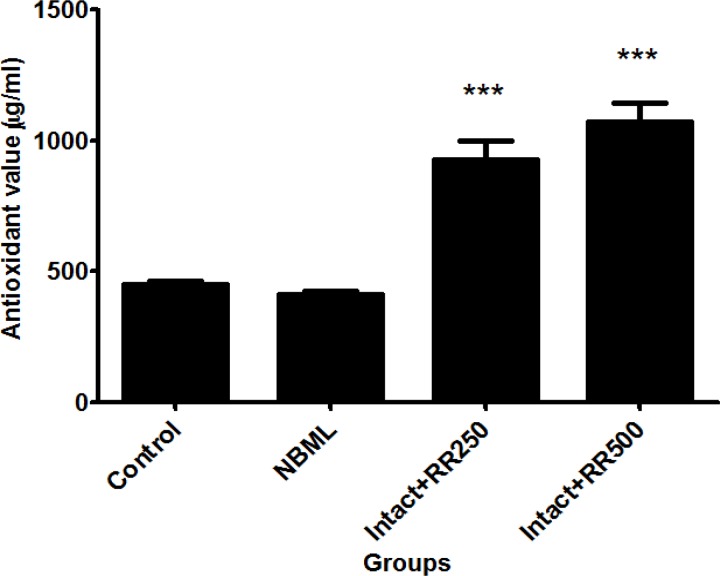
The antioxidant levels in control (450.6 ± 13.6), NBML (414.3 ± 13.7) and intact rats receiving *Rheum ribes *extracts (Intact + RR250 = 926.3 ± 73.1, Intact + RR500 = 1072 ± 70.1). The data are expressed as Mean ± SE; (n = 7 in each group).

Figure 3 shows the level of antioxidants in blood serum in control, NBML and both groups of intact rats receiving 250 and 500 mg/kg RR extracts. The level of antioxidants was markedly increased in Intact + RR250 and Intact + RR500 groups compared to the control group (p < 0.001).

*** p < 0.001 significant from control.

*The Effect of Rheum ribes Extract on Plasma level of Malondialdehyde (MDA)*

**Figure 4:**
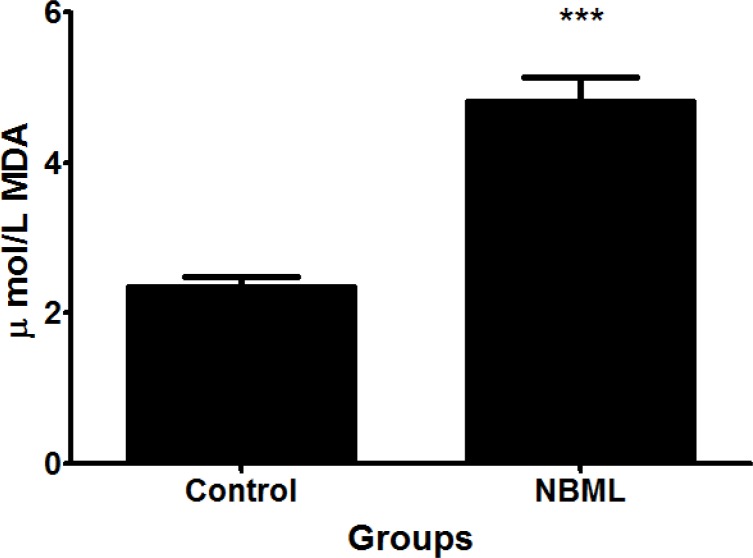
Malondialdehyde (MDA) levels in the control (2.35 ± 0.13) and NBML (4.81 ± 0.32) groups. The data are expressed as Mean ± SE; (n = 7 in each group).

Figure 4 compares the plasma level of malondialdehyde in the control and NBML groups. As seen in the Figure 4, MDA levels was significantly increased in NBML group in comparison with the control group (p < 0.001).

*** p < 0.001 significant from control.

In this research, we studied the effect of RR hydro-alcoholic extract on learning and memory in rat model of Alzheimer's disease, in which Nucleus Basalis of Meynert (NBM) was destroyed. The results of this study indicate that bilateral NBM lesions in rats lead to spatial memory deficits both in water maze and also in passive avoidance experiments. In addition, we examined the effect of hydro-alcoholic extracts of RR on memory impairments induced by NBM lesions. Our data demonstrate that RR extracts, in the concentrations of 250 and 500 mg/kg, improve memory deficit induced by NBM lesions in passive avoidance test as well as the water maze experiment. Furthermore; our results showed that serum MDA levels significantly increased in Alzheimer’s group compared to the control group. This high level of MDA is probably caused by NBM lesion.

Present findings regarding the effect of NBM lesion on memory is consistence with our previous studies (7, 31) in which it was shown that NBM lesions cause memory impairment. NBM nucleus is the primary source of cholinergic branches to hippocampus and cortex and plays a role in cognitive processes (32). Afferent cholinergic nerves extending from NBM to cortex is the pathway that is markedly compromised in brain of Alzheimer's patients (33). Cholinergic system is responsible for saving and retrieving information in memory. Destruction of cholinergic system in the brain is considered as the main reason for loss of memory and cognition in Alzheimer’s patients (34, 35). As most of the neurons in the NBM are cholinergic, it has been suggested that these reduction of cholinergic neurons may have role in the memory deficits observed in NBM-lesion rats.

It seems that increase in level of acetylcholine help vigilance, attention motivation and general activity of AD patients (36). It is also proved that high activity of acetylcholinesterase enzyme and therefore decrease in acetylcholine levels can cause loss of spatial memory and Alzheimer (37). Acetylcholinesterase inhibition is the most important strategy for the treatment of Alzheimer leading to increase the cholinergic function in the brain (38, 39). In one study it was revealed that Ginkgo biloba, which is used for the treatment of Alzheimer, has acetylcholinesterase inhibitory activity. It has been shown that flavonoid composition of this herb is responsible for its anti-acetylcholinestrase effect (40). In another study, Anderson reported that *Lavandula angustifolia* improves learning and memory through its anti-acetylcolinesterase activities (41). On the other hands, Gholamhoseini and colleagues demonstrated that RR has high acetylcholinesterase inhibitory activity (9). They found that IC50 for methanolic extract of this herb is 0.95 mg/ml. Therefore; it is possible that memory enhancement observed in present study is caused through anti-acetylcholinstrase activity of this herb.

Ozturk et al reported that RR root and stem methanolic extracts demonstrated high DPPH scavenging activity (18). On the other hands, it has been shown that antioxidants delay the progress of Alzheimer's disease probably due to prevention or neutralization of detrimental effects of free radicals (19). Our data indicate that RR extract has radical scavenging activity and also high level of antioxidants. Therefore, another possibility is that these radical scavenging activity and containing antioxidants are responsible for the beneficial effects of RR extract on passive avoidance and spatial memory.

Brain tissue is vulnerable to oxidative stress due to high demand of energy and oxygen. Increase in oxidative stress in elderly is a risk factor for Alzheimer disease (42). Epidemiological and laboratory evidences reveal that foods containing antioxidants can prevent the progress of Alzheimer disease, probably because of prevention or neutralization of destructive damage of free radicals (19). Studies on some of the foods and drinks, such as extract of garlic, melatonin, ginkgo biloba and foods containing vitamin C and vitamin E, have revealed their preventive and delaying effects on Alzheimer’s disease due to their antioxidant compounds (43). The results of present study showed that hydro-alcoholic extract of RR has high antioxidant activity which can have effect on reduction of memory disturbance of NBM lesion.

It has been shown that RR contains, flavonoid, quercetin, selenium and phenol (20, 21). In addition, Octay reported root ethanolic extract of *Rheum ribes* had higher phenolic content (44) which is consistent with our finding. Herbs containing flavonoid compounds have therapeutic effect on the diseases caused by oxidative stress (45). Quercetin is a flavonoid with high antioxidant characteristic, which also has anti-inflammatory feature (46). Some polyphenol compositions such as quercetin also pass through blood brain barrier and have protective effect against hydrogen peroxide (47). Selenium is an essential nutrient of diet. This micronutrient protects cells against damage of reactive oxygen species (ROS). In a study on Alzheimer patients by Cardoso, a relationship was found between oxidative stress in Alzheimer’s disease and selenium deficiency (48). Another study revealed that supplements containing selenium effectively improves cognitive deficiency, probably by dephosphorylation of tau protein (physiological factor of AD) (49). Putting all together it seems that RR extract rescue memory impairments induced by NBM lesions through its compositions such as flavonoids, antioxidants and its radical scavenging activity.

## Conclusion

In general, this study clearly revealed that treatment with *Rheum ribes* extract can markedly rescue spatial and passive avoidance memory impairments induced by destruction of NBM nucleus in rats. It suggests that this herb has potential therapeutic effects for neurological diseases, such as Alzheimer’s. Further investigation is needed to expand these findings.

*Conflict of interest statement*

We declare that there is no disclose any financial and personal relationships with other people or organizations that could inappropriately influence (bias) our work.
